# Supplementary figures and images for: Endolysosomal trafficking controls yolk granule biogenesis in vitellogenic Drosophila oocytes
Source: PLoS Genet. 2024 Feb 5;20(2):e1011152. doi: 10.1371/journal.pgen.1011152 (PMC10898735; doi:10.1371/journal.pgen.1011152)

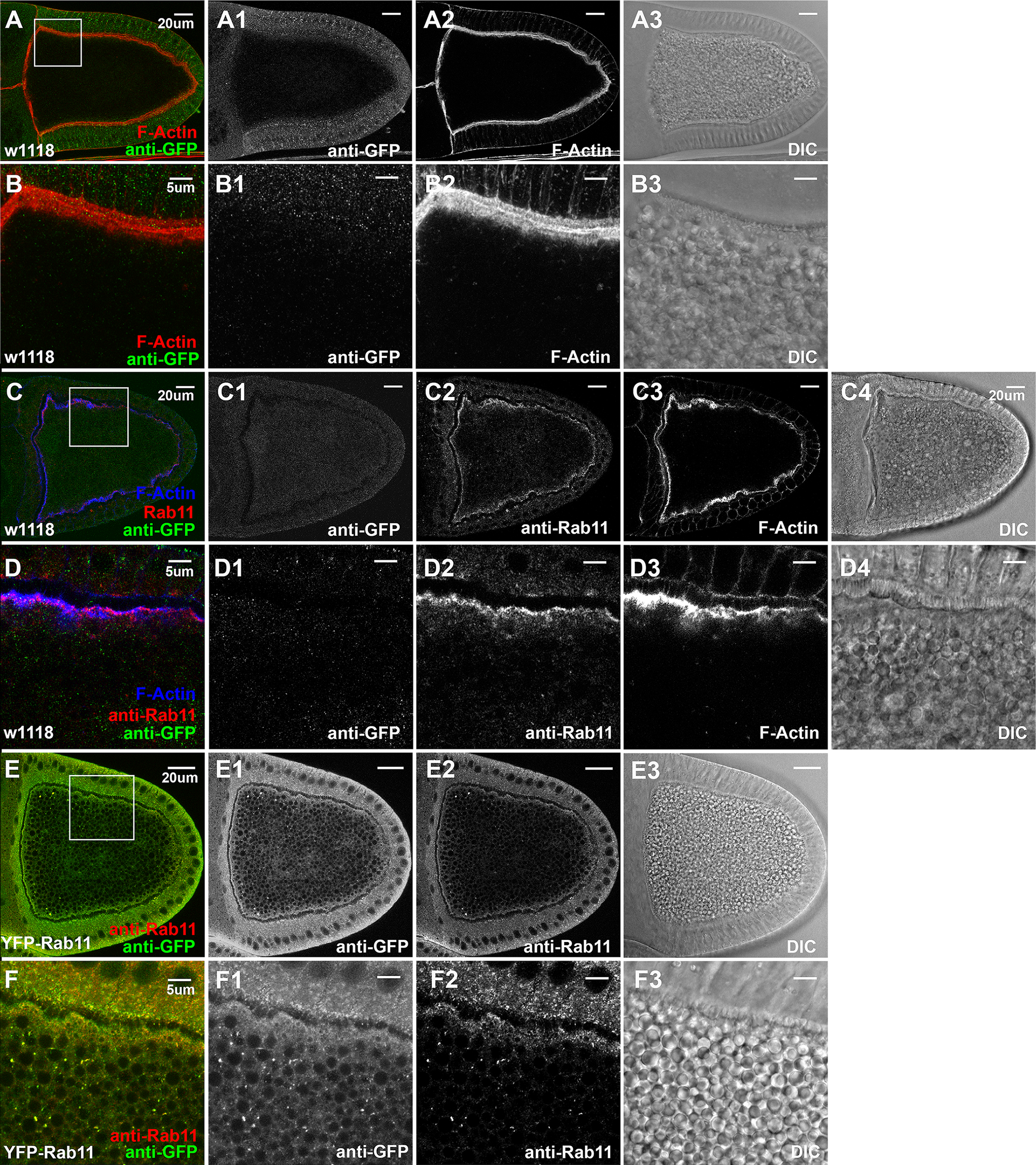

Supplement: S1 Fig — Confocal fluorescent microscopy and DIC imaging of stage 10 egg chambers with immunofluorescent staining by anti-GFP antibody (green) on (A-D) w1118 wild type control or (E, F) YFP-Rab11 endogenous tagging flies, co-labeled with (A-D) phalloidin for F-Actin and (C-F) mouse anti-Rab11 antibody, as annotated. (B, D and F) High magnification views of the regions highlighted in (A, C and E), respectively. Images are presented as overlaying in color or individual channels in gray, as annotated. Genotypes: (A-D) w1118; (E,F), w1118; TI{TI} EYFP-Rab11 (BDSC #62549). The sizes of the scale bars as annotated. (TIF) [file pgen.1011152.s001.tif]

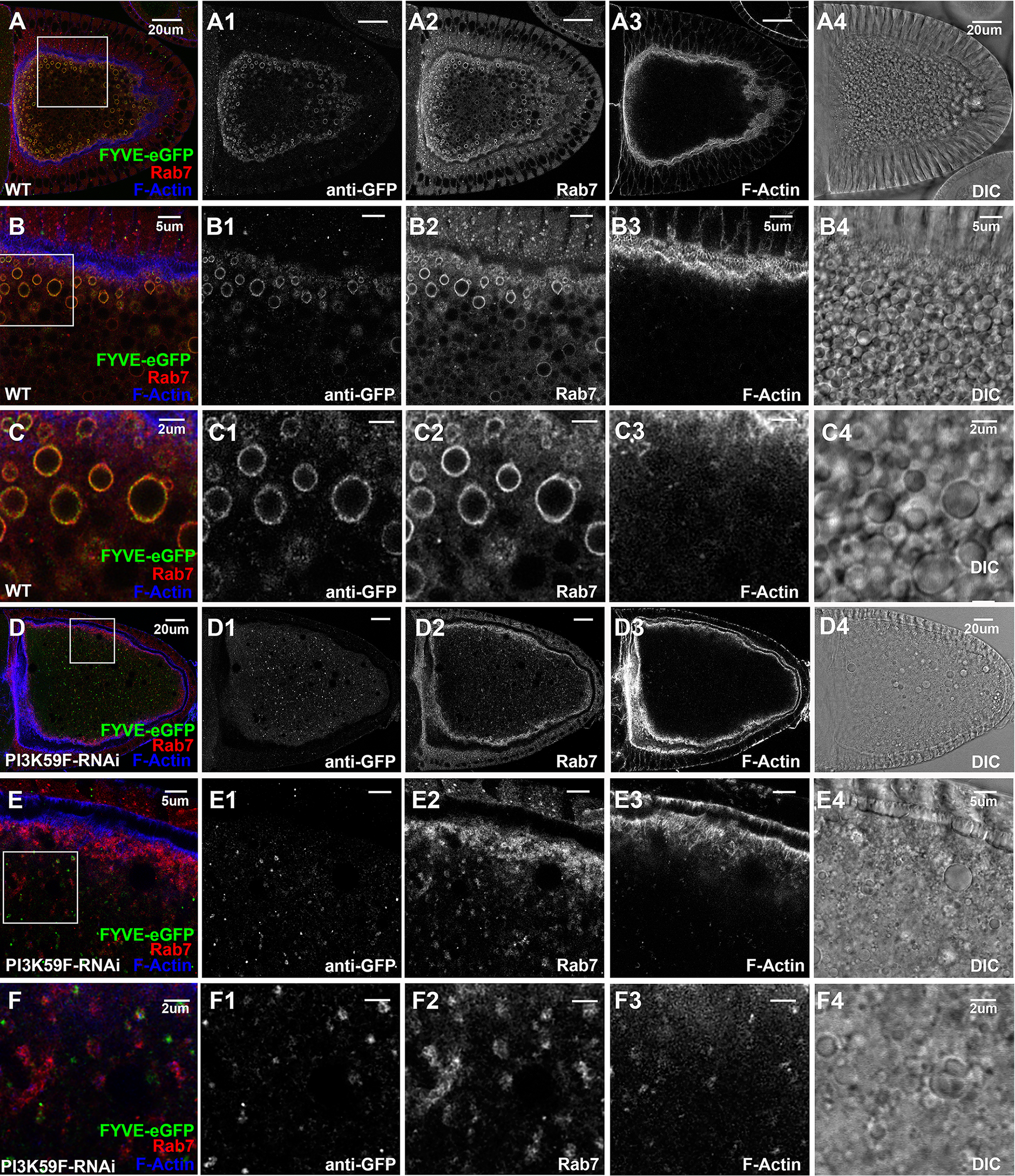

Supplement: S2 Fig — Confocal fluorescent microscopy and DIC imaging of stage 10 egg chambers with oocyte-specific expression of 2xFYVE-GFP reporter from (A-C) wild type control or (D-F) oocyte co-expressing dsRNA again VPS34/PI3K59F, triple-labeled for GFP (green), Rab7 (red) and F-Actin (blue). Images are presented as overlaying in color or individual channels in gray, as annotated. Genotypes: (A-C). w*/ w1118; matalpha4-GAL-VP16 (BDSC #7062), UAS-GFP-myc-2xFYVE (BDSC #42712)/+; (D-F). w*/ w1118; matalpha4-GAL-VP16 (BDSC #7062), UAS-GFP-myc-2xFYVE (BDSC #42712)/+; P{TRiP.HMJ30324}attP40 (BDSC #64011)/+. The sizes of the scale bars as annotated. (TIF) [file pgen.1011152.s002.tif]

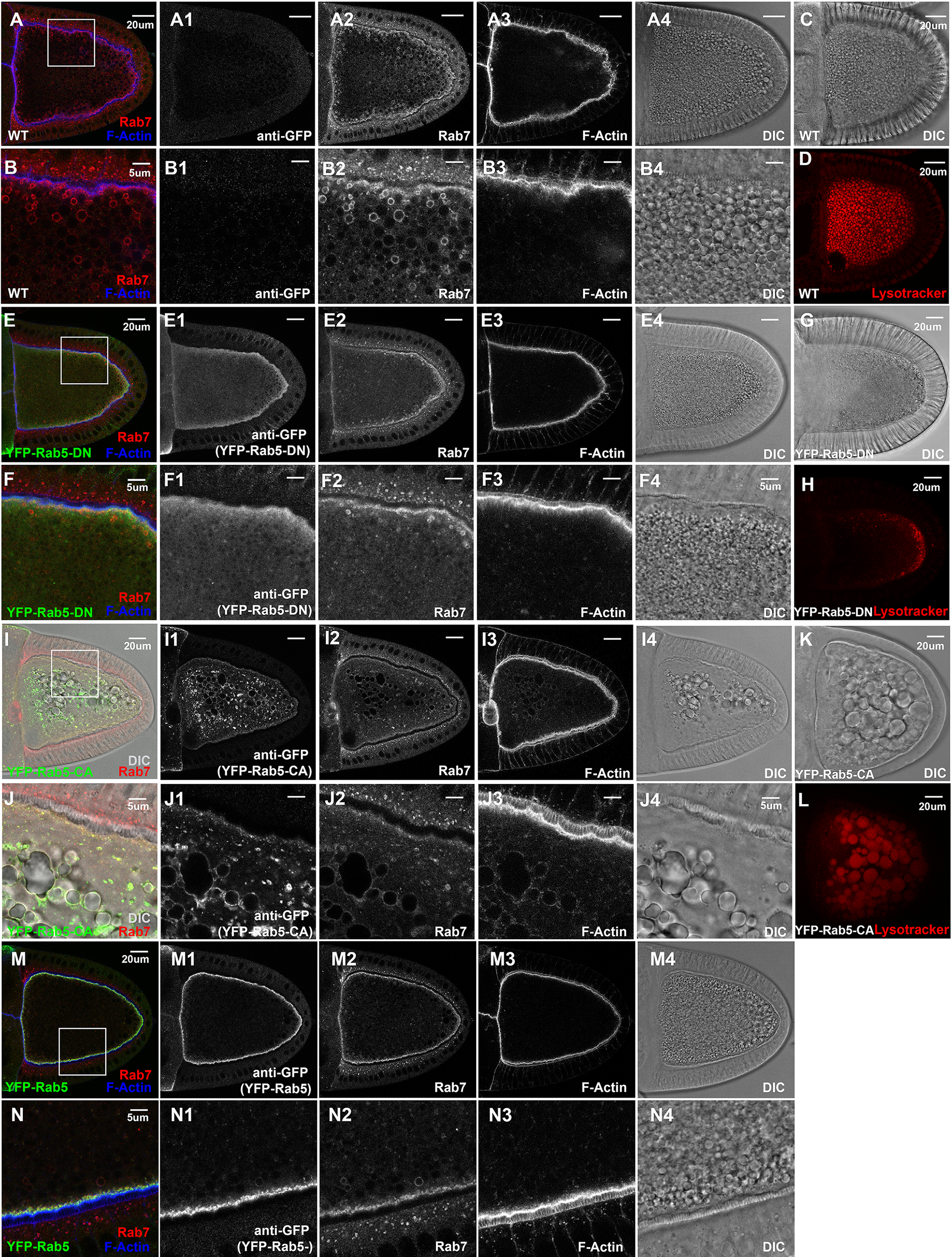

Supplement: S3 Fig — Confocal fluorescent microscopy and DIC imaging of stage 10 egg chambers from oocytes of (A-D) wildtype control or (E-N) overexpressing (E-H) dominant-negative YFP-Rab5-DN, (I-L) constitutive-active YFP-Rab5-CA and (M, N) wildtype YFP-Rab5, that were (A, B, E, F, I, J, M, N) triple-labeled for GFP (green), endogenous Rab7 (red) and phalloidin for F-Actin (blue), or (C, D, G, H, K, L) stained with lysotracker alone (red), with data presented as overlaying images in color or individual channels in gray, as annotated. (B, F, J and N) High-magnification views of the cortex regions highlighted in (A, E, I and M), respectively, as annotated. The sizes of the scale bars as annotated. Genotypes: The samples were from adult female flies heterozygous for both matalpha4-GAL-VP16 (#7062) driver and the following UAS-transgenic lines: (A-D) w1118. (E-H) Rab5-DN: P{UASp-YFP.Rab5.S43N}01 (#9771). (I-L). Rab5-CA: P{UASp-YFP.Rab5.Q88L} (#9774). (M, N). Rab5-WT: P{UASp-YFP.Rab5}02 (#24616). (TIF) [file pgen.1011152.s003.tif]

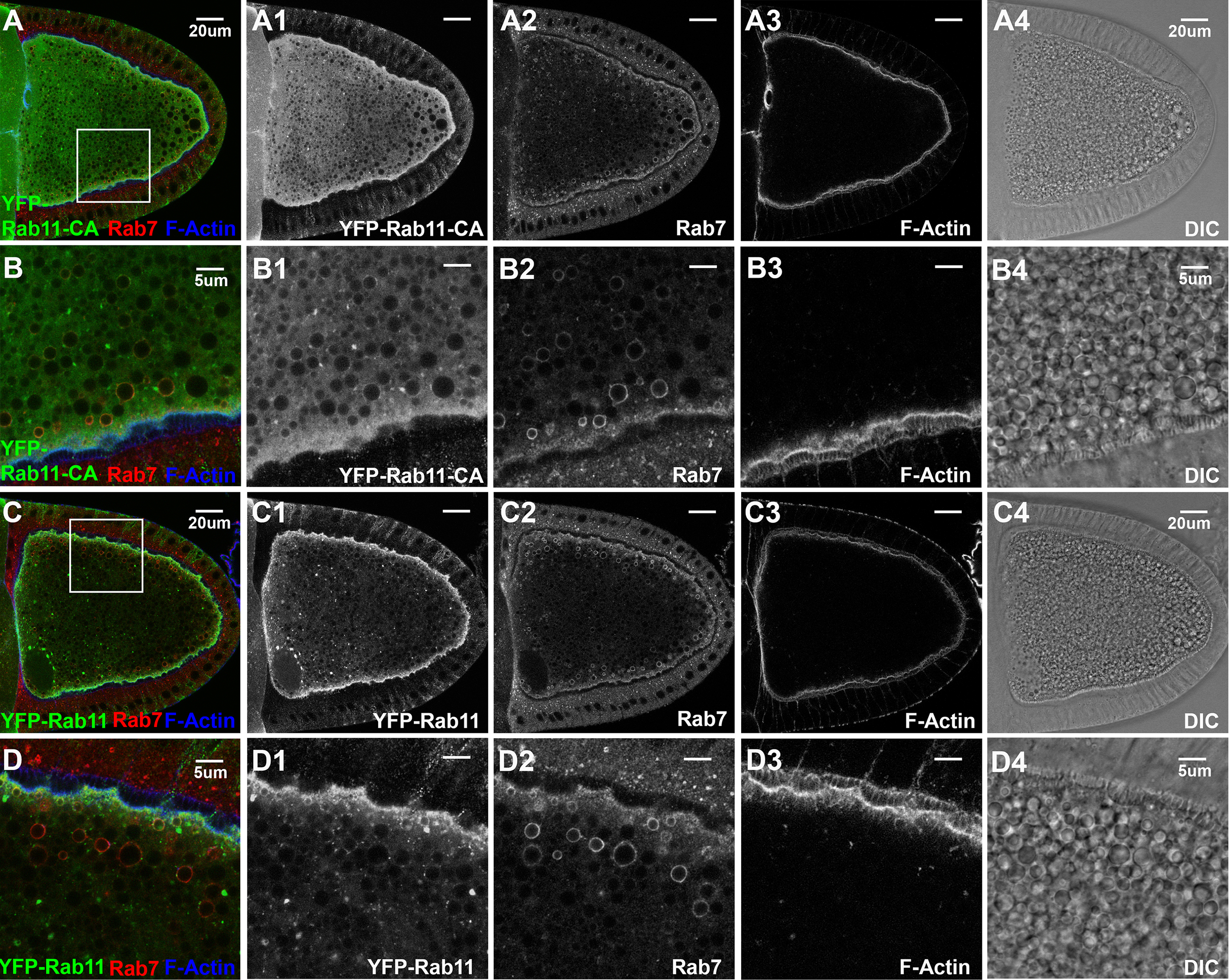

Supplement: S4 Fig — Confocal fluorescent microscopy and DIC imaging of a stage 10 egg chambers with oocyte-specific expression of (A, B) constitutive active YFP-Rab11-CA or (C, D) wildtype YFP-Rab11 triple-labeled for YFP (green), endogenous Rab7 (red) and phalloidin for F-Actin (blue), presented as overlaying image of all channels in color or individual channels in gray, as annotated. (B, D) High-magnification views of the cortex regions highlighted in (A, C), respectively, as annotated. Genotypes: The samples were from adult female flies heterozygous for both matalpha4-GAL-VP16 driver (#7062) and the following UAS-transgenic lines: (A, B) Rab11-CA: P{UASp-YFP.Rab11.Q70L} (#23260). (C, D). Rab11-WT: P{UASp-YFP.Rab11} (#50782). The sizes of the scale bars as annotated. (TIF) [file pgen.1011152.s004.tif]

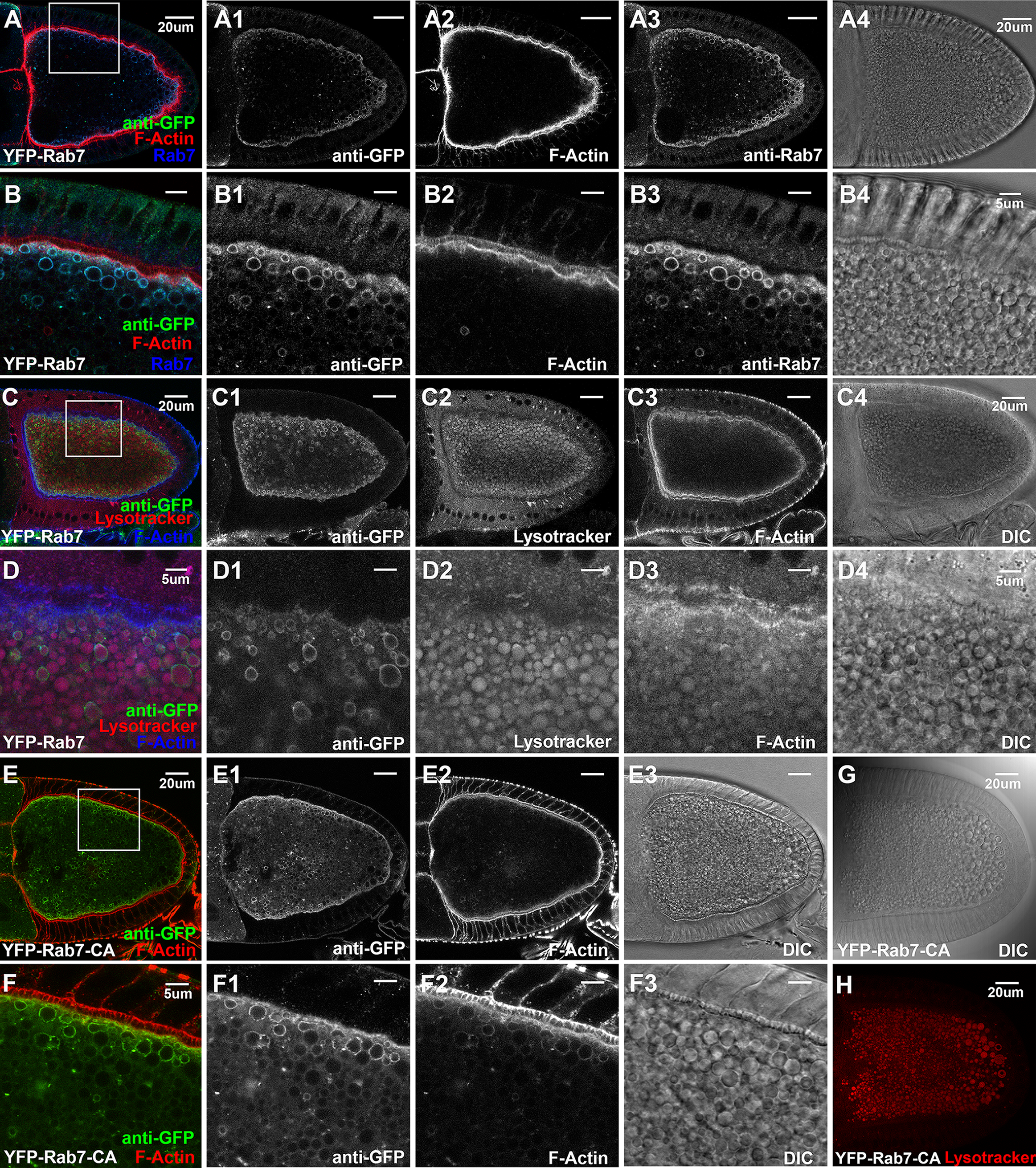

Supplement: S5 Fig — Confocal fluorescent microscopy and DIC imaging of stage 10 egg chambers with ectopic expression of (A-D) wildtype YFP-Rab7 or (E-H) constitutive active (CA) YFP-Rab7-CA in oocytes that are (A-F) co-labeled for anti-GFP (green), F-Actin and (A, B) anti-Rab7 or (C, D) lysotracker (red), or (G, H) by lysotracker (red) alone, shown as overlaying images of all the channels in color or in individual channels in gray, as annotated. (B, D, F) High-magnification view of the cortex regions highlighted in (A, C, E), respectively, as annotated. Genotypes: The samples were from adult female flies heterozygous for both matalpha4-GAL-VP16 driver (#7062) and (A-D) wildtype YFP-Rab7: y[1 w*; P{w(+mC) = UASp-YFP.Rab7}21/SM5 (#23641): (E, F) constitutive active YFP-Rab7-CA: P{UASp-YFP.Rab7.Q67L} (#24103).The sizes of the scale bars as annotated. (TIF) [file pgen.1011152.s005.tif]

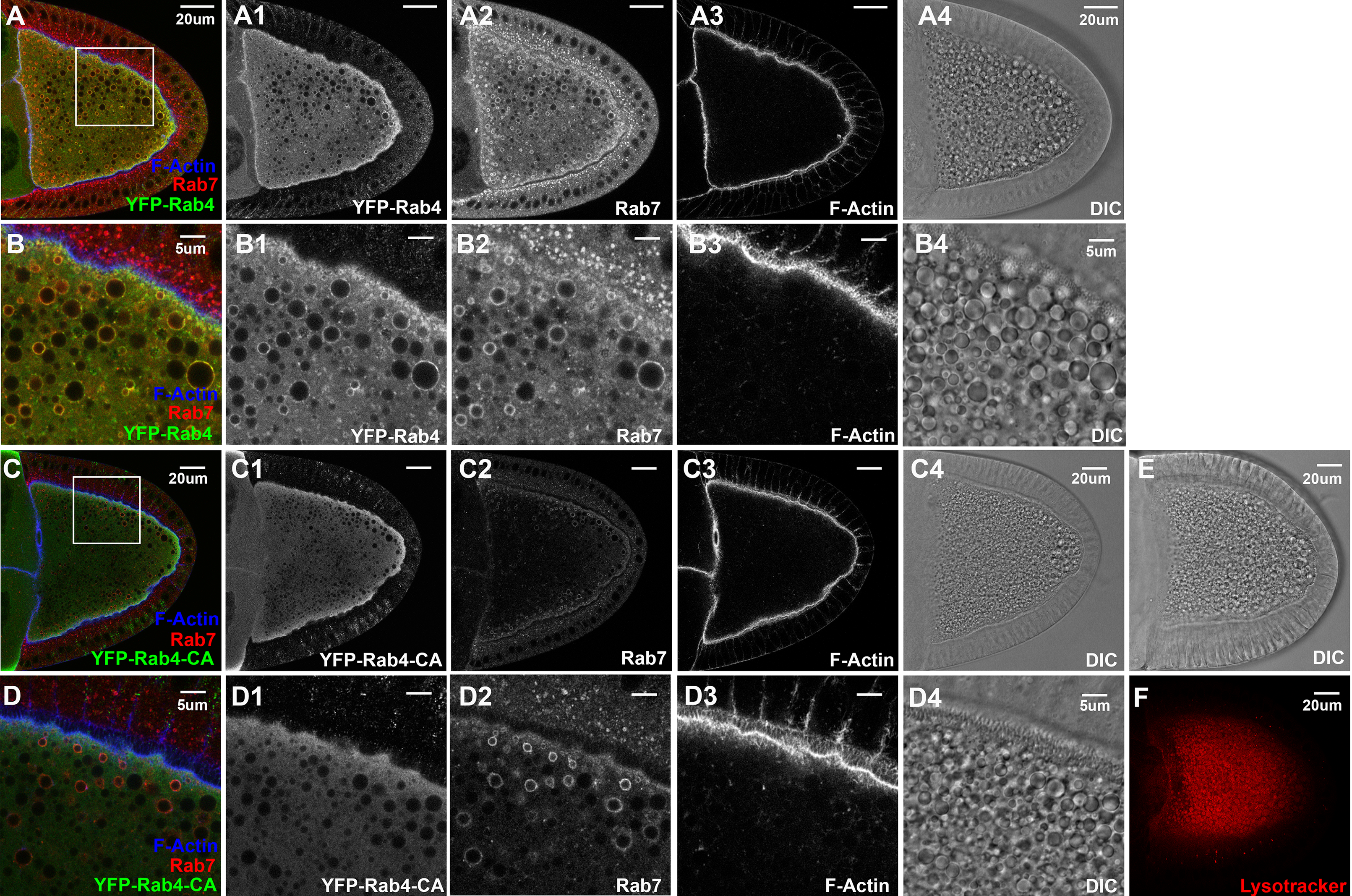

Supplement: S6 Fig — Confocal fluorescent microscopy and DIC imaging of stage 10 egg chambers with oocyte-specific expression of (A, B) wildtype YFP-Rab4 or (C-F) constitutive active YFP-Rab4-CA (A-D) triple-labeled for YFP (green), endogenous Rab7 (red) and F-Actin (blue), shown in overlaying images in color or individual channels in gray, or (E, F) by lysotracker staining (red) alone, as annotated. (B, D) High-magnification views of the cortex regions highlighted in (A, C), respectively, as annotated. Genotypes: The samples were from adult female flies heterozygous for both matalpha4-GAL-VP16 driver (#7062) and the following UAS-transgenic lines: (A, B) wildtype Rab4: P{UASp-YFP.Rab4} (#9767). (C-F) Rab4-CA: P{UASp-YFP.Rab4.Q67L} (#9770). The sizes of the scale bars as annotated. (TIF) [file pgen.1011152.s006.tif]

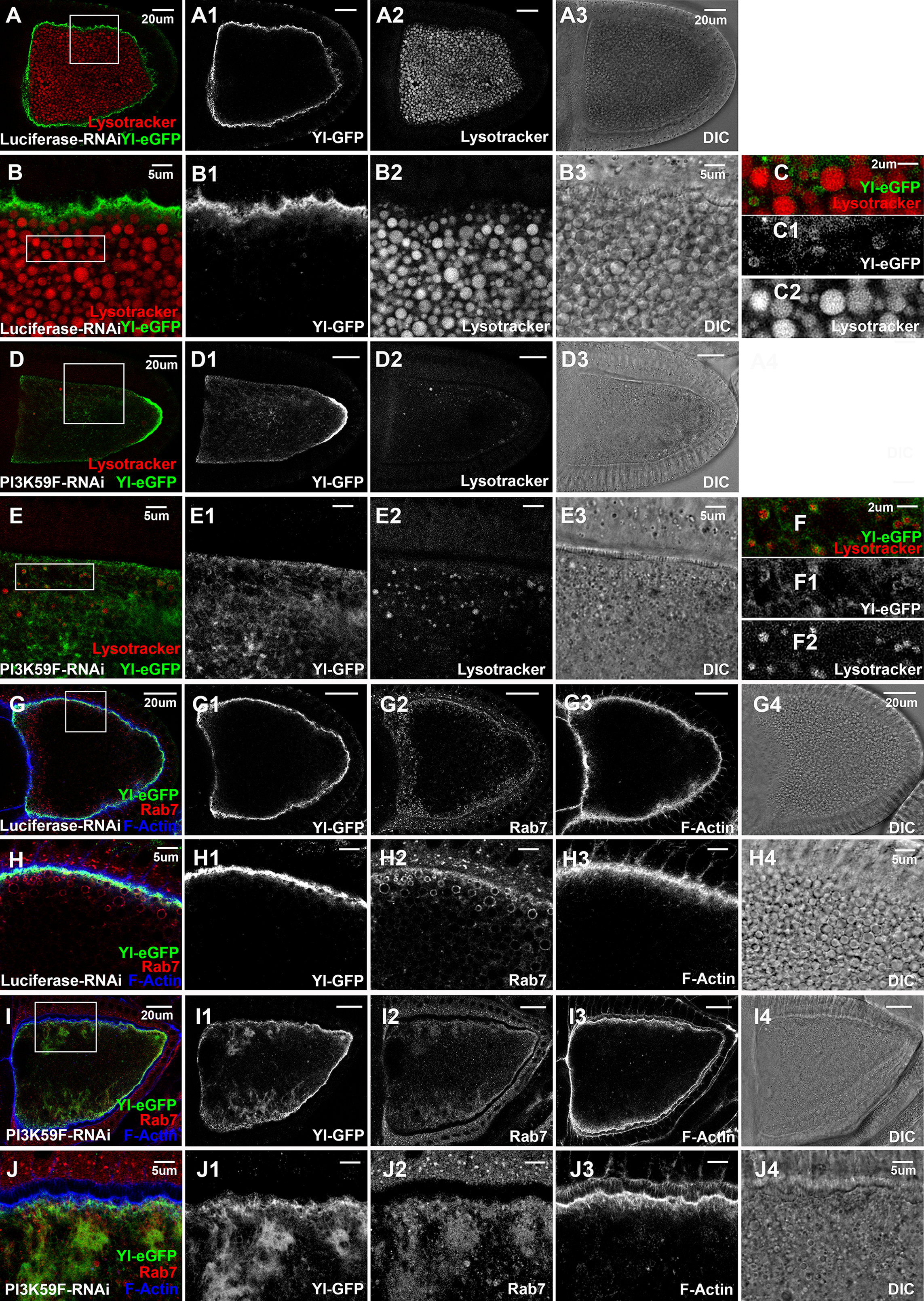

Supplement: S7 Fig — Confocal fluorescent microscopy and DIC imaging of stage 10 egg chambers from flies carrying genome-tagging Yl-eGFP-3xHA with oocyte-specific expression of (A-C, G, H) control firefly Luciferase RNAi or (D-F, I, J) dsRNA again VPS34/PI3K59F, co-labeled with antibodies against GFP (green) and (A-F) lysotracker (red), or (G-J) endogenous Rab7 (red) and F-Actin (blue), as annotated. (B, E, H, J) High-magnification view of the cortex regions highlighted in (A, D, G, I), respectively. (C, F) Zoom-in view of the areas highlighted in (B, E), respectively. Images are presented as overlaying images in color or as individual channels in gray, as annotated. Genotypes: The samples were from adult females flies heterozygous for Yl-eGFP-3xHA reporter (p{mini-W+, yl-eGFP-3xHA}) and matalpha4-GAL-VP16 (BDSC #7062) driver together with (A-C, G, H). P{TRiP.JF01355}attP2 (BDSC#31603) or (D-F, I, J) P{TRiP.HMJ30324}attP40 (BDSC #64011). The sizes of the scales as annotated inside images. (TIF) [file pgen.1011152.s007.tif]

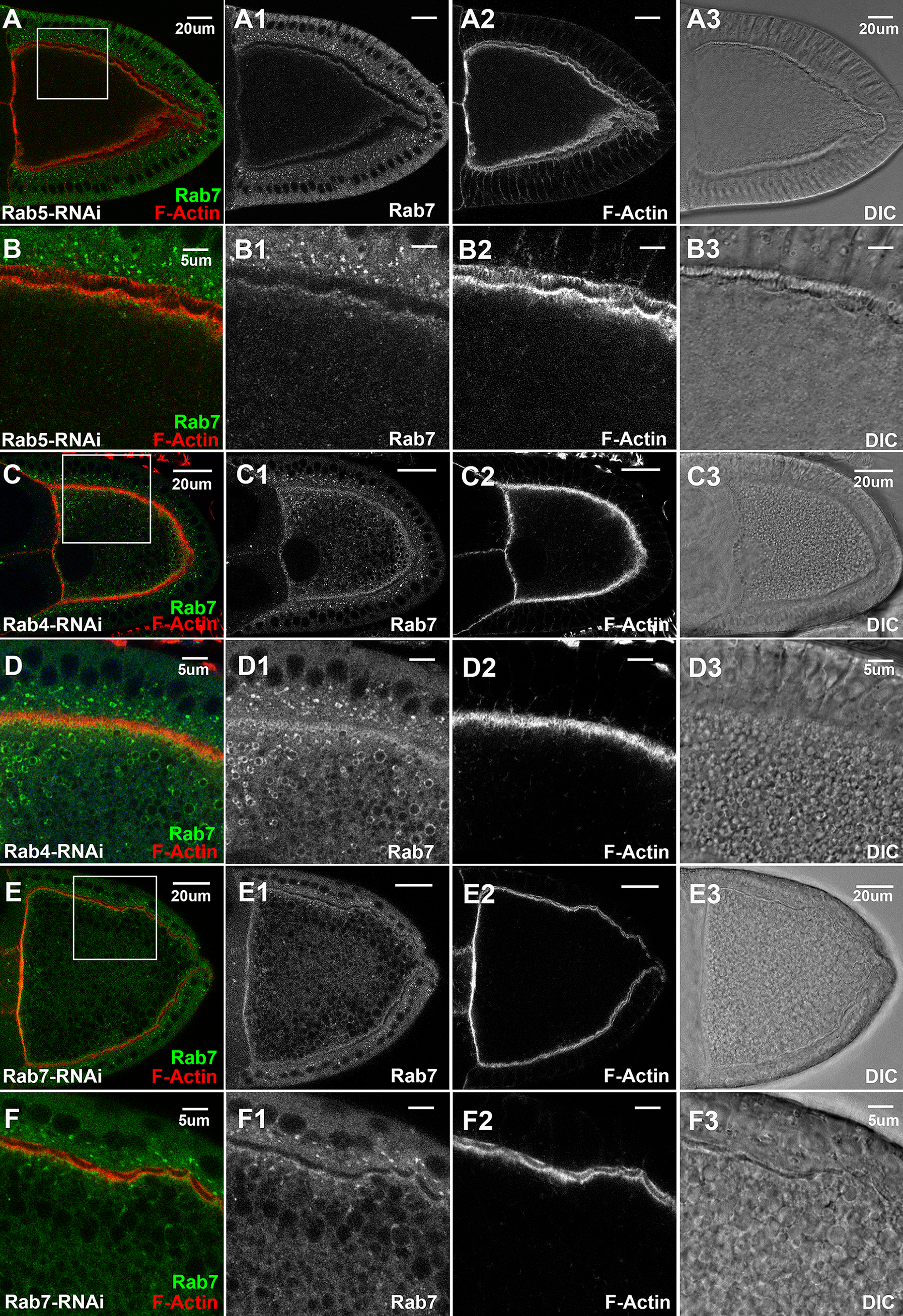

Supplement: S8 Fig — Confocal fluorescent microscopy and DIC imaging of stage 10 egg chambers with oocyte-specific expression of dsRNA against (A, B) Rab5, (C, D) Rab4 and (E, F) Rab7, co-stained for endogenous Rab7 (green) and F-Actin (red), as annotated. (B, D, F) High-magnification view of the cortex regions highlighted in (A, C, E), respectively. Images are presented as overlaying images in color or as individual channels in gray, as annotated. Genotypes: The samples were from adult females flies heterozygous for matalpha4-GAL-VP16 (BDSC #7062) driver together with (A, B) P{TRiP.GL01872}attP40 (BL# 67877). (C, D) P{TRiP.HMS01100}attP2P (BDSC #33757). (E,F) P{y(+t7.7] v(+t1.8) = TRiP.JF02377}attP2 (BDSC#27051). The sizes of the scales as annotated inside images. (TIF) [file pgen.1011152.s008.tif]
