# Supplementary material for: Endolysosomal trafficking controls yolk granule biogenesis in vitellogenic Drosophila oocytes
Source: PLoS Genet. 2024 Feb 5;20(2):e1011152. doi: 10.1371/journal.pgen.1011152 (PMC10898735; doi:10.1371/journal.pgen.1011152)
Supplement: Attachment — Submitted filename: Responses Letter -V2.docx [file pgen.1011152.s010.docx]

**Reviewer #1:**

 In this manuscript, Yu et al. shed light on the significance of Rab5, Rab11, Rab7, dynein motor, and the RAVE/V-ATPase complex in endocytosis, endosomal trafficking, and yolk granule biogenesis. The authors generated a tagged version of Yolkless (YI) and studied its distribution in vitellogenic oocytes. They observed a connection between different endosomal structures, phosphoinositides, and actin cytoskeleton dynamics. The study identified Rab5 and Rab11 as crucial players in yolk granule biogenesis, while Rab4 and Rab7 role was dispensable. The authors carried out a small RNAi screen on Rab5 effector molecules identified in a previous proteomic study and proposed the involvement of the dynein motor and the RAV/V-ATPase complexes in the early stages of endosomal trafficking.

The data presented in this manuscript offers novel insights into endolysosomal pathway, and highlight the vitellogenic oocyte in Drosophila as a valuable in vivo system to study endocytosis and endolysosomal trafficking. Such insights are of broad interest to researchers studying complex membrane trafficking events.

This is an exciting and clearly-written manuscript. The results are well organized, and the data support the interpretation and conclusions drawn. However, some revisions are recommended to improve the manuscript.

We thank the reviewer for the very supportive and constructive comments that our study “offers novel insights into endolysosomal pathway” and “Such insights are of broad interest …. This is an exciting and clearly-written manuscript”. We have revised the manuscript following reviewer’s comments.

1. To assess the functionality of the transgenic construct Yl-eGFP-3xHA, was it placed in yl mutant background?
Yes, we had carried out rescue experiment by introducing a 3^rd^ chromosome-located p[Yl-eGFP-3xHA] transgene into a strong *yl* loss of function allele *yl^13^* (BDSC #4320), which is homozygous sterile. The presence of p[Yl-eGFP-3xHA] transgene completely rescued the female sterile phenotypes of *yl^13^* adults. The homozygous *yl^13^* flies (genotype: *yl^13^*; p[Yl-eGFP-3xHA]) are being maintained as a stable stand-alone stock.

Following the reviewer’s suggestion, we have included the description of the rescue experiment into the revised manuscript.

2. For readability, it would be beneficial to combine Figure-1 and Figure-2 (Inserting Figure-2 panel between Fig.1D and Fig.1E)
We had combined the Figure 1 and Figure 2 into **revised Figure 1**.

3. In Fig.1G, please provide an inset of invaginating clathrin-coated pits/vesicles if available.

We had included insets of invaginating clathrin-coated pits and vesicles into **revised Figure 1J**.

4. Please cite this article “Crystalline yolk spheroids in Drosophila melanogaster oocyte: Freeze fracture and two-dimensional reconstruction analysis” (<https://doi.org/10.1016/j.jinsphys.2006.12.011>) when referring to the condensed and crystalized yolk protein in mature granules.

We had added the paper as reference **#57** to the revised manuscript. We thank the review for this information.

5. Briefly describe how the transgenic construct Yl-eGFP-3xHA was used to rescue the sterility of yl mutant females.
We had added the description of the rescue experiments and the related fly stock information into the revised manuscript.

6. It would be beneficial to place an arrow in the figures when referring to F-actin puncta (E.g. Fig.4B and 4I)
Arrows were added to highlight the F-actin puncta in **revised Fig 3(B, D, I**).

7. What were the selection criteria for selecting specific Rab5 effectors for the RNAi screens?
In the RNAi screen, we selected the Rab5 effectors reported in Supplemental Table-1 from the Gillingham et al. study (“Toward a Comprehensive Map of the Effectors of Rab GTPases” (2014) Dev Cell *31*, 358-373. 10.1016/j.devcel.2014.10.007).

We mainly chose the ones with relatively low “Frequency” number in Supplemental Table-1 of the Gillingham study, as the low “Frequency” number should indicate relatively high specificity of the isolated effectors for active Rab5 than for other Rab proteins. Additionally, given the limited scope of our study, we only selected the candidates with existing TRiP RNAi lines from Bloomington Stock Center, which were designed to have high knockdown efficiency and minimal off-target effect.

As the Reviewer 3 recommended to shorten and condense the manuscript, we did not include the above information on the selection criteria for the tested Rab5 effectors in the revised manuscript. But we will be happy to include them if the Reviewer think it would help the manuscript.

8. RNAi lines might have a variable knockdown efficiency and potential off-targets, since most of the tested lines did not show any effect on granule biogenesis in the RNAi screen

We fully agree with reviewer’s insightful comment on potential drawbacks related to RNAi-mediated studies, including inefficient knockdown and off-target effect. Indeed, we believe that in our study, a significant number of the RNAi lines with no clear phenotypes were possibly due to the low knockdown efficiency of the tested RNAi lines.

We also tested RNAi lines for Rab4, Rab5 and Rab7 (**new Fig S8)**, and found that as expected, RNAi against Rab4 did not show apparent effect on granule biogenesis, Rab5 RNAi induced strong phenotypes very similar to, or even more severe than, that by Rab5-DN dominant negative, although RNAi line against Rab7 only partially depleted endogenous Rab7 (**new Fig S8**), which are in line with reviewer’s comment on the variable knockdown efficiency by RNAi lines (One side note is that we also tried RNAi for Rab11, but the cross using the RNAi line against Rab11 (BDSC stock #27730) failed to produce progenies for unknown reason despite repeated effort).

In the second to the last paragraph of the revised manuscript, we **added an extended discussion** on this important point raised by the reviewer.

was qPCR performed to the test the knockdown efficiency?
We have not done qPCR to test the knockdown efficiency, mainly because of the experimental design that makes the evaluation by qPCR not feasible.

In the study, we used matalpha4-GAL-VP16 to specifically knockdown target gene expression in the germline-derived oocyte and nurse cells, but not in other cell types and tissues including the surrounding follicle cells and muscles that ensheathed the oocyte. Because of this, the isolated egg chambers will always be a mixed population of cells, with dsRNA being expressed only in oocytes and nurse cells but not in the surrounding follicle cells and muscles. Because it is not possible to separate these surrounding cells from the oocyte in the same egg chambers, qPCR would measure transcripts from a mixed population of cells, therefore the knockdown effect in the oocyte would be masked by the normal levels of transcripts in the surrounding follicle and muscle cells that do not express the dsRNA.

As briefly discussed in the manuscript, the reasons we used the matalpha4-GAL-VP16 to drive dsRNA expression are that the oocyte-specific expression allows us to study essential genes, whose global depletion would cause animal lethality and prevent their functional evaluation for granule biogenesis. For example, when directed by the ubiquitous Tubulin-GAL4, we found that RNAi lines against Rab5, PI3K59F and Vps15 and several other genes caused animal lethality.

In addition, for some essential genes, their depletion from earlier oocyte stages would block oocyte development or cause other secondary effects that interfere with the later granule biogenesis starting from stage 8 (analyzed in stage 10 in our study). Because matalpha4-GAL-VP16 initiates dsRNA expression around stage 5 of egg chamber development, it should bypass this early functional requirement of the studied gene while still leave sufficient times for RNAi-mediated knockdown of target genes by stage 10 when the egg chambers were analyzed in our study, in particular for effective RNAi lines.

However, this relatively limited duration between stage 5 and stage 10 might not be sufficient for RNAi lines that are less effective or for proteins that are more stable with long half-life, which might explain our observations of incomplete knockdown by RNAi line against Rab7 (**new Fig S8**).

Nevertheless, overall we found that matalpha4-GAL-VP16 line was able to produce robust and specific phenotypes by several RNAi lines we tested, including Rab5 and PI3K59F/Vps15 and Rbcn-3A, as described in the manuscript.

In future, we plan to continue to optimize the RNAi conditions for vitellogenic oocytes, including to test additional oocyte-specific GAL4 lines for more reliable and robust RNAi-mediated depletion of target genes in this tissue, and to explore the new CRISPR/gRNA approach for loss of function studies in vitellogenic oocytes.

Again, we thank the reviewer for the very insightful comments.

9. It would be great if the authors can clarify the number of replicates performed to validate the observed phenotype in the RNAi screens, either in the figure legend or the methods section.
We have added the following description in the revised method “Dissection and immunofluorescent staining and imaging of egg chambers” section.

“Each experiments were repeated independently at least once. In each experiment, at least 5 adult females of appropriate genotypes were dissected for immunofluorescent staining and microscopy imaging, 20 or more stage 10 egg chambers were inspected under immunofluorescent microscope, and five or more independent egg chambers were imaged through Z-serial section by confocal microscope.”

10. The study focuses on stage 10 oocytes, and the findings might be specific to this developmental stage. Independent of the context and potential redundancy it would help readers to discuss the general applicability of the findings in other cell types, tissues, or organisms.

We fully agree with this important point. Following the suggestion, we **added a brief discussion** in the last paragraph of the revised manuscript to highlight the limit of our study only on stage 10 oocytes and the need for more studies on oocytes of other stages and on other cell types, tissues and organisms.

**Reviewer #2:**

In their manuscript Yu and colleagues are showing a detailed microscopic analysis of the endocytic pathway that is essential for the generation of the yolk granules in the Drosophila oocyte. The authors use multiple Rab transgenes and Rab5 effector RNAis to genetically dissect the sequence of the events of this pathway. Although the study is comprehensive and aims to give a more complete view about this phenomenon, it still has multiple flaws, like it relies almost entirely on one technique (confocal microscopy) but lacking any quantifications and contains multiple formal mistakes. Thus, the manuscript requires substantial improvement before it may get considered for acceptance in this Journal.
We thank the reviewer for commenting that “the study is comprehensive” and for the many constructive suggestions.

Major comments:
1. The first and most important flaw is the complete lack of quantifications and statistics. As the novelty factor of the findings is moderate the comprehensiveness could the major advantage of the study. However, it is really hard to compare the effect of the different genetic modifications without any quantifications. For example, visually the phenotype upon loss of of Rbcn3 may seems similar to the silencing of the class III PI3K complex, but it is really hard to make any conclusions without statistics.
We thank the reviewer for this very important point. We fully agree that quantification and statistics will greatly enhance the comparability among different genotypes, a goal we would like to reach.

We also like to explain that although we had tried, but we found it was very difficult to establish a quantification standard that could be applied uniformly to all the genotypes in this study, given the significant phenotypic differences and variations among different genotypes. The parameters used in the study, as summarized in the Supplemental Tables, were limited and mostly *descriptive*, such as the subcellular localizations of Yl receptor as well as Rab7 and F-Actin patterns, and the presence or absence of recognizable granules under confocal imaging.

Because the primary goal of this study is to examine if yolk granule biogenesis follows the canonical endolysosomal trafficking pathways, we only focused on the major players in the pathways and on the RNAi lines that produced consistent and strong phenotypes, not the lines with relatively minor and more variable effect.

Overall the results support vitellogenic oocyte as an useful in vivo model to study endolysosomal trafficking, although they did raise several questions that warrant further investigations in the future, especially to develop reliable quantification measures that allow a more accurate evaluation on the effect of studied genes in yolk granule biogenesis, as the Reviewer suggested.

In future studies, we plan to test additional endosomal markers and carry out more detailed analyses, in order to define parameters that can be used for reliable quantification of oocyte phenotypes at different endosomal trafficking steps, and to establish a uniform quantification standard in this unique in vivo model, an important future goal we aim to achieve together with input from colleagues in the field.

2. The authors are using the overexpression of DN forms of the Rab proteins to show their loss of function phenotype. However, RabDN-s are frequently cause weaker loss of function phenotype than mutant alleles or RNAi-s. So, it would be important to confirm the effect of DN lines by silencing of Rab4, Rab5, Rab7 and Rab11 by appropriate RNAi lines.
Following the reviewer’s comment, we tested RNAi lines for Rab4, Rab5, Rab7 and Rab11 (**new Fig S8**). For unknown reason, the cross for RNAi line against Rab11 (BDSC stock #27730) with matalpha4-GAL-VP16 did not produced progenies despite repeat experiments. For others, we found that as expected, Rab4 RNAi did not apparently affect granule biogenesis, Rab5 RNAi induced strong phenotypes very similar to, or even more severe than, that by Rab5 dominant negative, although RNAi line against Rab7 only partially depleted endogenous Rab7 (**new Fig S8**), which again support potentially variable knockdown efficiency by different RNAi lines.

We agree with the reviewer that Rab-DNs often cause weaker phenotype than mutant alleles. The reason we primarily used DN is that Rab-DNs have been well-established for loss of function studies, and in our experience, they also produced more consistent (albeit might weaker) loss of function phenotypes, while RNAi lines were more variable in their knockdown effects, as insightful observed by the Reviewer in the Point #3 comment below.

Because of these considerations, in this study, we primarily used RabDN to evaluate the loss of function effect of these Rab proteins.

3. In the data from the small-scale screen on Rab5 effectors (Table S1) it is visible that in some cases the used RNAi lines that silence the same gene (like in the case of Vps15) showed different phenotypes. Was the effectivity of RNAi lines tested (like with qPCR)?
Indeed, as discussed in comment Point 2 above, our data showed the variable knockdown efficiency by different RNAi lines against the same gene.

We have not done qPCR to test the knockdown efficiency, because the experimental design made it not feasible to use qPCR to evaluate the RNAi knockdown efficiency in the oocyte.

Briefly, in the study, we used matalpha4-GAL-VP16 driver to specifically knockdown target gene expression in the germline-derived oocyte and nurse cells, but not in other cell types including the surrounding follicle cells and muscles that ensheathed the oocyte. Because of this, the isolated egg chambers will always be a **mixed** population of cells, with dsRNA being expressed only in oocytes and nurse cells but not in surrounding follicle cells and muscle cells. Because it is not possible to separate these surrounding cells from the oocyte in the same egg chambers, qPCR would measure total transcript levels from this mixed population of cells, and any knockdown effect in the oocyte would be masked by the normal levels of transcripts from the surrounding follicle and muscle cells that do not express the dsRNA.

As briefly discussed in the manuscript, the reasons we used the matalpha4-GAL-VP16 driver for RNAi study are that its oocyte-specific expression allows us to study essential genes, whose global depletion cause animal lethality and prevent their functional examination in granule biogenesis. For example, when directed by the ubiquitous Tubulin-GAL4, we found that RNAi lines against Rab5, PI3K59F, Vps15 and several other genes caused animal lethality.

In addition, for some essential genes, their depletion from earlier oogenesis stages will block oocyte development or cause other secondary effects that would interfere with the granule biogenesis starting on stage 8 (analyzed in stage 10 in our study). Because matalpha4-GAL-VP16 initiates dsRNA expression around stage 5 of egg chamber development, this would bypass earlier functional requirement of the studied gene while still leave sufficient times for RNAi-mediated knockdown of target genes by stage 10 when the egg chambers were analyzed in our study, especially for effective RNAi lines.

However, the relatively limited duration between stage 5 and stage 10 might not be sufficient for RNAi lines that are less effective or if the targeted proteins are more stable with long half-life, which might explain the incomplete knockdown efficiency by RNAi line against Rab7 (**new Fig S8**) that we observed, and the variable knockdown effect by different RNAi against the same gene (e.g., Vps15), as noted by the Reviewer.

Nevertheless, overall we found that this matalpha4-GAL-VP16 line was able to produce robust and specific phenotypes for several RNAi lines we tested, as described in the manuscript (e.g., Rab5, PI3K59F/Vps15, Rbcn-3A).

In the future, we plan to continue to optimize the RNAi conditions in the vitellogenic oocytes, including to test additional oocyte-specific GAL4 lines to achieve more reliable and robust RNAi-mediated depletion of target genes in this tissue, and also to explore the new CRISPR/gRNA method for loss of function studies in the vitellogenic oocytes.

4. Authors sometimes claim interesting suggestions based on fluorescent mictoscopy data, but these are remaining as speculations without testing them by molecular methods. For example, based on the results with ctp RNAi Authors suggest that the dynein-dynactin complex would work as the effector of Rab5 instead of Rab7 in this tissue. Although this is an attractive idea it should be tested by pull down or immunoprecipitations.
We fully agree with the reviewer’s comment. Currently we do not have validated anti-Ctp antibody for the pull down experiment to further test this hypothesis. Review 3 also suggested to remove this Ctp data from the manuscript, as it does not directly relate to the main goal of the study.

Given the similar comments from both reviewers on this issue, we had removed the ctp RNAi data in the revised manuscript.

5. Upon the knockdown of PI3K59F (Fig6C) it is clearly visible that remarkable amount of FYVE-GFP positive structures. Which is surprising, as loss of PI3K59F should diminish the formation of PI3P positive granules. Was the RNAi efficient enough?
We believe the remaining FYVE-GFP positive structures were likely due to incomplete depletion of PI3K59F protein in the oocyte.

As discussed in more details for Points 2 and 3 raised by the Reviewer, due to the experimental design in our study, RNAi driven by the tissue- and stage-specific matalpha4-GAL-VP16 likely will not completely deplete target proteins in the oocyte by stage 10. Due to a lack of specific antibody for PI3K59F, currently we were not able to directly test the knockdown efficiency by RNAi on endogenous PI3K59F protein.

We also found that global depletion of PI3K59F by RNAi, driven by stronger and ubiquitously expressed Tubulin-GAL4, caused animal lethality. In this study, RNAi by the oocyte-specific matalpha4-GAL-VP16 allowed us to study PI3K59F’s role in granule biogenesis, although it might not induce complete loss of function phenotypes for PI3K59F in the oocyte.

What can be the identity of these persistent FYVE-GFP granules? Are these persistent FYVE granules are colocalizing with the remaining Rab7 puncta?

Following the reviewer’s comment, we did more detailed studies (please see **new Fig S2D-F** in the revised manuscript). The data suggested that the persistent FYVE granules most likely were related to remnants of early endosomal structures, as they co-aggregate together with Rab7 in the same areas inside the oocytes, but the two only partially overlap on the same structures (please see **new Fig S2F**), unlike that in wildtype control (please see revised **Fig S2C**) where Rab7 and FYVE showed significant co-localization on the same granules).

6. Fig6A shows that upon the KD of PI3K59 YL-GFP is leaving the cortical region and delivered further inside into the oocyte. Does YL-GFP is reaching the lysosomes upon these conditions? Although the overlap between the diffuse signal of YL-GFP and Rab7 is apparent, but there are still some Rab7 and YL-GFP puncta remaining in these cells. Are these YL-GFP puncta colocalizing with Rab7 or lysosomal markers?

To address the reviewer’s question, we performed co-labeling and more detailed imaging of YL-GFP with lysotracker and with Rab7 in wildtype and PI3K59F-RNAi oocytes.

The data showed that Yl-GFP puncta is unlikely to reach the lysosomes (**new Fig S7A-F**), and although Yl-GFP and Rab7 showed quite similar pattern of sequestration in PI3K59F-RNAi oocytes, they only partially overlapped (**new Fig S7J**). It appeared that Yl-GFP and Rab7 were trapped in the same loci but might not be within the identical structures, or they might be accumulating in different loci within the same abnormal membranous structures. More studies are needed, especially using super-resolution microscope in the future, to fully address this interesting question.

We have included these new data in new **supplemental Figure S7** in the revised manuscript.

Interestingly, in wildtype oocyte, detailed imaging revealed that YL-GFP often surrounded the small-sized lysotracker-positive granules, but not the larger ones (**Fig S7C**). Similarly, in PI3K59F-RNAi oocytes, YL-GFP also surrounded the few remaining small lysotracker-positive granules (**Fig S7F**). This could suggest that depletion of PI3K59F/VPS34 did not interrupt the earlier step of endosomal trafficking, or this is simply due to the activity by the remaining PI3K59F in the RNAi-expressing oocytes. Future studies are needed to clarify this interesting question.

We thank the Reviewer for these insightful observations and comments.

7. On Fig9 the Authors are using the silencing of two Vha genes to show the effect of loss of the V-Atpase on yolk granule formation. However, they are using different RNAi lines to show the effect on Rab7 (Vha26 RNAi), and FYVE-GFP (Vha68 RNAi). Although the phenotypes are consistent, to rule out any concern of pleiotropic effects it would be better to use the same RNAi for both markers (or use both RNAis for both).
We have included Rab7 data by Vha68-2 RNAi in the **revised Figure 8** in the manuscript.

Minor comments:
1. The Legends for main figures and supplementary figures and sometimes the figures itself contains multiple inconsistence and annoying mistakes:

We deeply apologize for these negligence, and sincerely thank the reviewer for pointing out these mistakes. We have gone through the manuscript multiple times and hoped have fixed all the mistakes.

- Fig1: What are the green circles that appear on panels E, F, G? In the legend the second reference for panel (F) actually refers to panel (G)

The “Oo” in panels E, F, G stand for “oocyte. We have changed “Oo” to “oocyte” in the **revised Fig 1**. We thank the reviewer for noticing the mislabeling on panel (F) and have corrected it.

- Fig4: The UASp-PLCD1(PH) reporter is called as PLCD-eGFP in the legend, but PH-GFP is written on the image panel, this inconsistence should be resolved. The legend refers to panel (G) that shows FYVE-GFP, however on the image this panel shows PH-GFP

We corrected the errors in the revision (**revised Fig 3**).

- FigS3-S5: The logic of the panel labels of FigS3-S5 is different from the logic that was followed by the rest of the Figures. This inconsistence should be resolved.

We have revised the panel labels in Fig S3-S5 (**Fig S4-6 in the revised manuscript**) to the same format as for the other Figures.

- FigS5: The order of panel labels restarts after the 10th panel (panel J followed by another A, B, C, etc.) and inconsistent with the legend. This should be corrected.

We have revised the panel labels for FigS5 (**Fig S6 in the revised manuscript**).

2. On the 18th page of the submitted PDF Authors are writing the following: “overexpressing either DN-, WT- or CA-YFP-Rab7, as large numbers of lysotracker-positive yolk granules were present in these oocytes (Fig 5C-F and Fig. S4)”, however there are no panel showing the YFP-Rab7WT on these figures.

We have added YFP-Rab7WT data to the **revised Fig S5A, B**, and also include new data for co-labeling between YFP-Rab7WT and lysotracker (**Fig S5C, D**). We apologize for the negligence and thank the reviewer for pointing this out.

3. There is no reference for Fig7E, F panels in the main text

We apologize for the negligence. Fig 7E, F were data related to Ctp knockdown, which is removed in the revised manuscript.

**Reviewer #3:** Endocytic recycling is a fundamental cellular process that plays a key role in all tissues, and yet it has mostly been studied in mammalian tissue culture cells or yeast. The Drosophila model system has a lot of potential as a means to investigate how endocytic processes vary between tissues and are regulated to meet their disparate needs.

The Rab GTPases are known to be master regulators of the endocytic pathway, and this paper reports an analysis of the role of key Rabs in the endocytosis of vitellogenin and yolk granule formation in developing oocytes. These maturing oocytes are very large and so amenable to imaging, and also accessible with tools such as ectopic protein expression and RNAi for following cellular processes and perturbing protein expression. The authors apply these methods to investigate the contributions that Rabs 4, 5, 7 and 11 make to the endocytosis and recycling of Yolkless (the vitellogenin receptor) and the formation of yolk granules. They also investigate the effect of knocking down a set of c 16 putative Rab5 effectors that were identified in a previous study based on affinity chromatography. Overall, the authors conclude that Rab5 and Rab11 play key roles in the process and identify several Rab5 effectors that are particularly important. They also find that Rab7 is not critical for Yolkless recycling and granule formation which is an unexpected finding but convincingly shown by both the Rab7 reagents and the knock-down of a Rab7 GEF. This raises interesting questions about what purpose Rab7 serves in such recycling pathways in tissues.

The paper describes a lot of work, and the data consists almost entirely of a large number of immunofluorescence images which are of a good quality and are clearly presented with both lower magnification overviews and higher magnification images to show details. The text is clear, although it would benefit from condensing and also some grammatical tidying. Overall, the paper makes a useful contribution to the endocytosis field as it shows the potential of the oocyte system, and in particular its value for studying Rab5 and Rab11. However, before publication the following relatively minor issues would need to be addressed:

We thank the reviewer for the very supportive and constructive comments that our study “are of a good quality and are clearly presented” and “makes a useful contribution to the endocytosis field”.

a) The text is rather long in the Introduction, Results and Discussion. Being more succinct would encourage more people to read the paper. It would be good to get the input from someone with lots of paper writing experience.
Following the suggestion, we have tried our best to shorten the Introduction, Results and Discussion and correct the grammar errors. We thank the reviewer for the very constructive suggestion.

b) For most of the figures the single IMF channels are shown as grey scale which makes them easier to see than colour. However, this is not the case for Figure 3, and so this should be altered to follow the better practice of the other figures.
We have changed the single channels for Figure 3 (**revised Fig 2**) to grey scale. We have made similar changes to other colored figures.

c) The authors state in the Introduction that PI3P acts as a landmark to enlist Ccz1/Mon1, but as they state later it is actually Rab5 that does this. There are other PI3P binders that could be mentioned instead.
We revised the text and cited EEA1 and Hrs proteins as two examples of PI(3)P binders. We thank the reviewer for this constructive suggestion.

d) For figures that show a YFP-Rab, the authors should include the YPF in the labelling on the figure and not just the name of the Rab to make clear that it is a tagged protein (ie YFP-Rab5 rather than Rab5).
We have added the YFP in the labeling for YFP-tagged Rab proteins in all figures.

e) Did the authors look at the distribution of Rab11 in an oocyte that is not also expressing YFP-Rab11? If so, it should be shown.
We have included data for Rab11 distribution in wildtype un-tagged oocytes, detected by mouse anti-Rab11 antibody staining, in the **revised Fig S1 (Fig S1C, D)**.

f) In Figure S2 they could include a wild-type control showing Rab7 and actin so as to aid comparisons. Perhaps the figure could be split into two figures to facilitate this.

We have included wildtype controls stained for Rab7, F-actin and also lysotracker in Figure S2 (now as **Fig S3A-D in Revised Fig S3**).

g) Figure 5D is labelled Rab-DN but it should be Rab7-DN.
We corrected this error (**revised Fig 4D**).

h) The authors should mention that flies lacking Rab4 are viable and fertile (in contrast to the situation for the other Rabs that they investigate). See PubMed ID 33666175.
We included the Rab4 knockout results and cited the paper as **reference #76**.

i) The section on cut up (ctp) could be deleted as it is not directly linked to the Rab work in the rest of the paper.
We removed the ctp data from the revised manuscript.

k) It would be helpful to have a summary table showing the effect on the various markers of all of the different Rab perturbing treatments tested.

We included a new **Table-S1** that summarizes the effect of perturbations by overexpressing wildtype and mutant Rab proteins in the oocytes.
