# Supplementary material for: Endolysosomal trafficking controls yolk granule biogenesis in vitellogenic Drosophila oocytes
Source: PLoS Genet. 2024 Feb 5;20(2):e1011152. doi: 10.1371/journal.pgen.1011152 (PMC10898735; doi:10.1371/journal.pgen.1011152)
Supplement: Attachment — Submitted filename: Responses-PLoSG-Decision (PGENETICS-D-23-00857R1).docx [file pgen.1011152.s011.docx]

**Reviewer #1:**

The authors have done an excellent job of addressing and responding to all the comments and concerns raised during the review process. They have diligently offered well-reasoned explanations and additional data where necessary to support their findings and conclusions.

We deeply thank the reviewer for the consistent support with the comments that “The authors have done an excellent job” and that “have diligently offered well-reasoned explanations”.

**Reviewer #2:**

The Authors addressed most of my concerns experimentally. I also approve their arguments regarding the difficulties of the quantification. This problem was further addressed by the use of a large number of samples (20< oocytes/genotype). Overall, the data and the manuscript's quality greatly improved.

We are grateful for the reviewer’s very supportive comments of “approve their arguments regarding the difficulties of the quantification... Overall, the data and the manuscript's quality greatly improved.”

I still have one minor comment: The texts "Yl" and (DIC) on Fig1 G1 and G2 have been mixed up. This should be corrected.

We have corrected this error in the revision. We apologize for this negligence and thank the reviewer for pointing it out.

**Reviewer #3:**

In revising this manuscript the authors have done an excellent job of addressing the points that I made. It was already a good quality study and I am now very happy to recommend that it be published. I have one minor suggestion which is that the newly added Table S1 seems a valuable summary of all the data which would help readers. Thus it might be good to incorporate it into the main paper rather that include it as a supplementary Excel file.

We deeply thank the reviewer for the very supportive comments that “I am now very happy to recommend that it be published”.

Following the reviewer’s suggestion, we have incorporated the Table S1 to the main paper as Table 1, and changed the label for Table S2 to Table S1 accordingly.
